# Discovery of Novel Caeridins from the Skin Secretion of the Australian White's Tree Frog, *Litoria caerulea*

**DOI:** 10.1155/2018/8158453

**Published:** 2018-07-11

**Authors:** Lei Li, Qing Wu, Xi Wang, Huimin Lu, Xinping Xi, Mei Zhou, Chris J. Watson, Tianbao Chen, Lei Wang

**Affiliations:** ^1^Natural Drug Discovery Group, School of Pharmacy, Queen's University, Belfast BT9 7BL, UK; ^2^School of Chinese Materia Medica, Beijing University of Chinese Medicine, Beijing 102488, China; ^3^Centre for Experimental Medicine, School of Medicine, Dentistry and Biomedical Sciences, Queen's University, Belfast BT9 7BL, UK

## Abstract

Abundant biologically active peptides have been discovered from frog skin secretions, a rich natural source of bioactive compounds with great potential in drug discovery. In this study, three Caeridin peptides, namely, Caeridin-1, S5-Caeridin-1, and Caeridin-a1, were discovered from the skin secretion of the Australian White's tree frog, *Litoria caerulea*, for the first time, by means of combining transcriptomic and peptidomic analyses. It also represents the first report on bioactive Caeridins since this family of peptides was initially studied 20 years ago. Chemically synthetic versions of each natural Caeridin demonstrated promising bioactivities either on rat smooth muscles or against microbial growth. Specifically, Caeridin-1 produced contraction of rat bladder smooth muscle, while S5-Caeridin-1 induced relaxation of rat ileum smooth muscle, both at nanomolar concentrations. Moreover, Caeridin-a1 was shown to potently inhibit the growth of the planktonic Gram-positive bacteria *Staphylococcus aureus* (*S. aureus*), methicillin-resistant *S. aureus* (MRSA), and *Enterococcus faecalis* (*E. faecalis*), the Gram-negative bacterium, *Escherichia coli* (*E. coli*), and the yeast, *Candida albicans* (*C. albicans*). The discovery of these Caeridins may induce further intensive and systematic studies of frog skin peptides to promote the discovery of natural templates as lead compounds for drug discovery and therapeutic application.

## 1. Introduction

In recent years, peptide and peptide-based drugs have been developing fast and have pioneered an essential area in the pharmaceutical industry [1]. Peptide-based medicinal therapy could offer benefits over other biologics, while demanding less costs in production [1]. Recent trends suggest that the application of therapeutic peptides is becoming more popular, and it has extended to the treatment of many diseases, such as infection, cancer, enzyme deficiency disorders, protein-dysfunction disorders, and even genetic and degenerative diseases [2].

Frog skin with poisonous glands can produce secretions in a holocrine manner and release complex chemical contents [3–5]. On the one hand, peptides and proteins contained in these secretions have been considered to play different but important roles in regulating frogs' physiological functions and defense against predators and microorganisms. On the other hand, they have been proven to hold hidden treasures to supplement drug development [6]. However, with the observed global decline in the frog population during the 1980s, including species extinction, it became apparent that this potentially valuable natural resource needed environmental protection and animal conservation to ensure peptide discovery and acquisition from these natural products could continue and play a role in the future development of novel therapeutic peptides [7, 8].

The Australian White's tree frog, *Litoria caerulea* (*L. caerulea*), belongs to the tree frog family Hylidae. Most members of this tree frog family can produce multiple defensive and regulatory chemical compounds in their skin secretions to provide protective survival advantages in their natural environment. Compared with the peptide discovery research on other tree frogs, previous studies on the skin secretions of *L. caerulea* were not as productive in terms of peptide quantities acquired and in the limited identification of peptides with a range of bioactive properties. The original study investigating peptides derived from *L. caerulea* was conducted in 1993 by Waugh et al. [9] who reported the discovery of 25 peptides from the secretions of the parotoid glands, including Caerulein 1, eighteen Caerins, and six Caeridins (Caeridins 1–6), the latter also being evidenced later in four other Australian frog species, including, *L. gilleni*, *L. splendida*, *L. xanthomera*, and *L. chloris*. In 1997, Steinborner et al. [10] reported the discovery of Caeridin 7.1 from the dorsal glandular skin extract of the brown tree frog, *Litoria ewingii*. Interestingly, with the Caeruleins showing hypotensive bioactivity and the Caerins antimicrobial activity, the Caeridins, however, were not reported to display any structural and functional resemblance to the Caerulein and Caerin peptides. These small Caeridin peptides (about 1000–1500 Da), comprising of 12–15 amino acid residues, share the same sequence -Gly-Leu-Leu/Phe- at the N-terminal ends and -Leu/Ile (NH_2_) at the C-terminal ends. Each of this Caeridins may be well fitted into an *α*-helical wheel with clearly delineated hydrophilic and hydrophobic zones [9]. The possession of dominant hydrophobic zones may suggest appreciable hydrophobic moments of these Caeridins and consequentially may bind more easily to the surface of biological membranes [11]. However, despite their distinct structural features, none of the Caeridins was reported to have any bioactive functions within these previous studies, which might explain why there has been no research reported on Caeridins over the last 20 years. Here, we report the isolation and structural characterisation of Caeridin-1 and two novel Caeridins from the skin secretion of *L. caerulea*, which were obtained through an ecofriendly method described previously [12], using parallel transcriptomic and peptidomic analyses, as well as through examining their biological activities using synthetic versions in a variety of bioassays.

## 2. Results

### 2.1. “Shotgun” Cloning of cDNAs Encoding Caeridins

**Figure 1:**
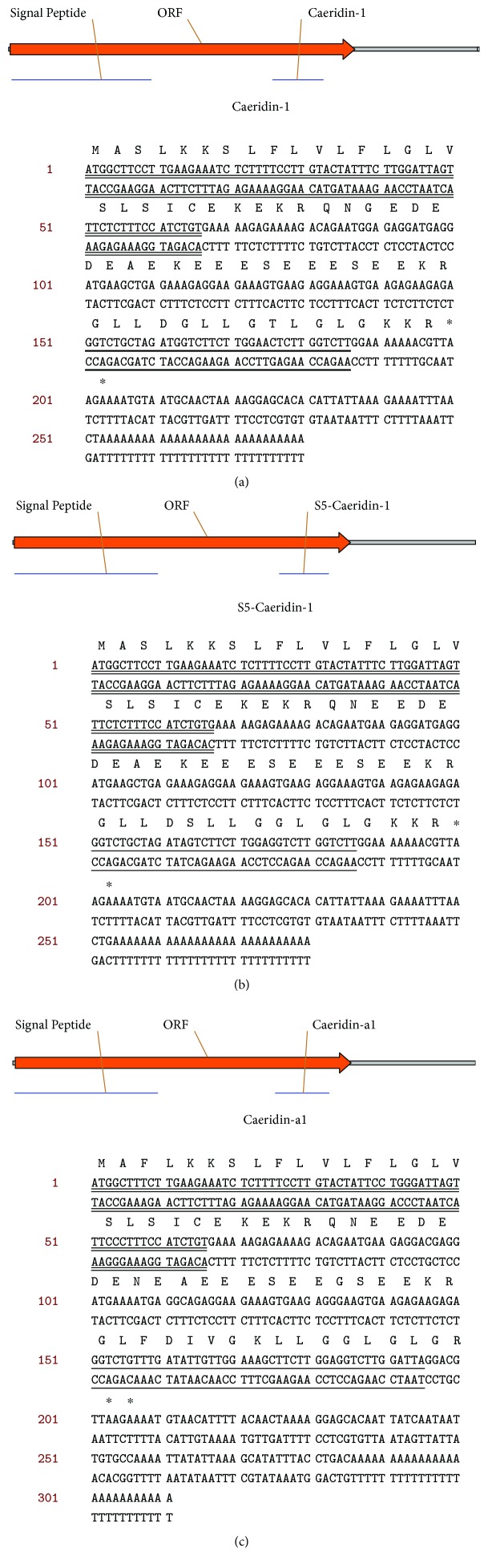
Nucleotide sequences of full-length cDNA clones encoding open-reading frames of (a) preprocaeridin-1, (b) preproS5-caeridin-1, and (c) preprocaerdin-a1 cloned from a *Litoria caerulea* (*L. caerulea*) skin secretion cDNA library. Putative signal peptide sequences are double-underlined, mature Caeridin sequences are single-underlined, and stop codons are indicated by asterisks.

**Figure 2:**
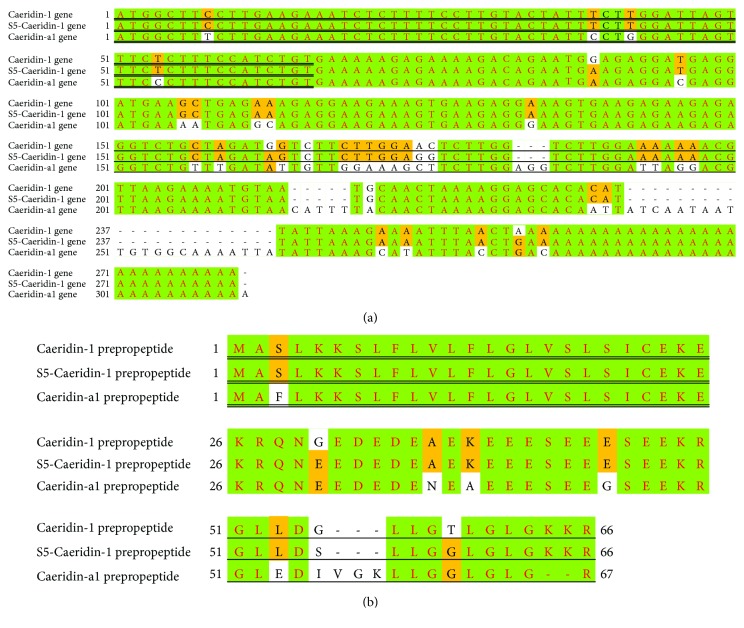
Alignments of (a) nucleotide sequences of cloned preprocaeridin cDNAs and (b) translated preprocaeridin open-reading frames established MEGA6. The identical and conservative regions are highlighted in green and yellow, respectively. Putative signal peptide sequences encoding regions and putative signal peptide sequences are double-underlined, and mature Caeridin sequences encoding regions and mature Caeridins are single-underlined.

**Figure 3:**
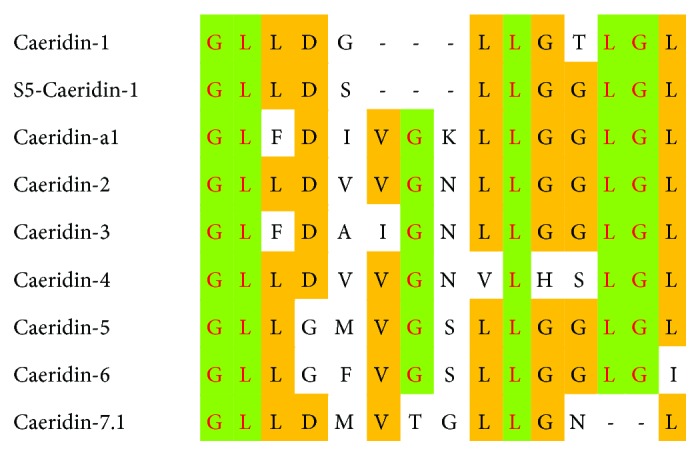
Alignments of amino acid sequences of Caeridins identified from *L. caerulea*. The identical and conservative amino acids were highlighted in green and orange, respectively.

Preprocaeridin cDNAs were consistently cloned from the skin secretion cDNA library, and each encoded a single copy of Caeridin, which were named Caeridin-1, S5-Caeridin-1, and Caeridin-a1 shown in Figure 1. The putative signal peptide sequences were localized to the acidic spacer peptide and were highly identical in structure. The latter encoding regions were flanked at the N-terminus by classical propeptide convertase-processing sites involving a propeptide convertase with -Lys-Arg- (-KR-) cleavage specificity, which suggested that there were mutations in the structure. Alignments of the full-length nucleic acid sequences and the translated open-reading frame amino acid sequences are shown in Figure 2 and highlight that preprocaeridins exhibit high degrees of both nucleotide and peptide primary structural similarities and conservation. Transcripts of Caeridin-1, S5-Caeridin-1, and Caeridin-a1 were registered with and deposited at http://www.ebi.ac.uk/ena/data/view/ permanently under the accession numbers LT852758, LT852759, and LT853865, respectively. Caeridin-1, S5-Caeridin-1, and Caeridin-a1 were compared with other Caeridins isolated from *L. caeulea*, and their amino acid sequences showed certain degrees of similarities suggested by the amino acid alignment in Figure 3.

### 2.2. Fractionation and Primary Structural Identification of Caeridins

**Figure 4:**
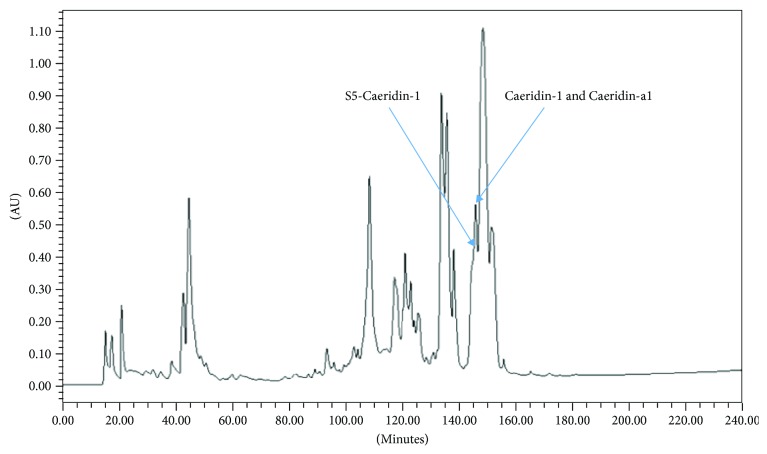
Reversed-phase high-performance liquid chromatography (HPLC) chromatogram of the skin secretion from *L. caerulea*. Caeridin-1, S5-Caeridin-1, and Caeridin-a1 were eluted at approximately 144 min, 143 min, and 144 min, respectively. The *y*-axis shows the relative absorbance in absorbance units (AU) at a wavelength of 214 nm, and the *x*-axis shows the retention time in minutes.

**Figure 5:**
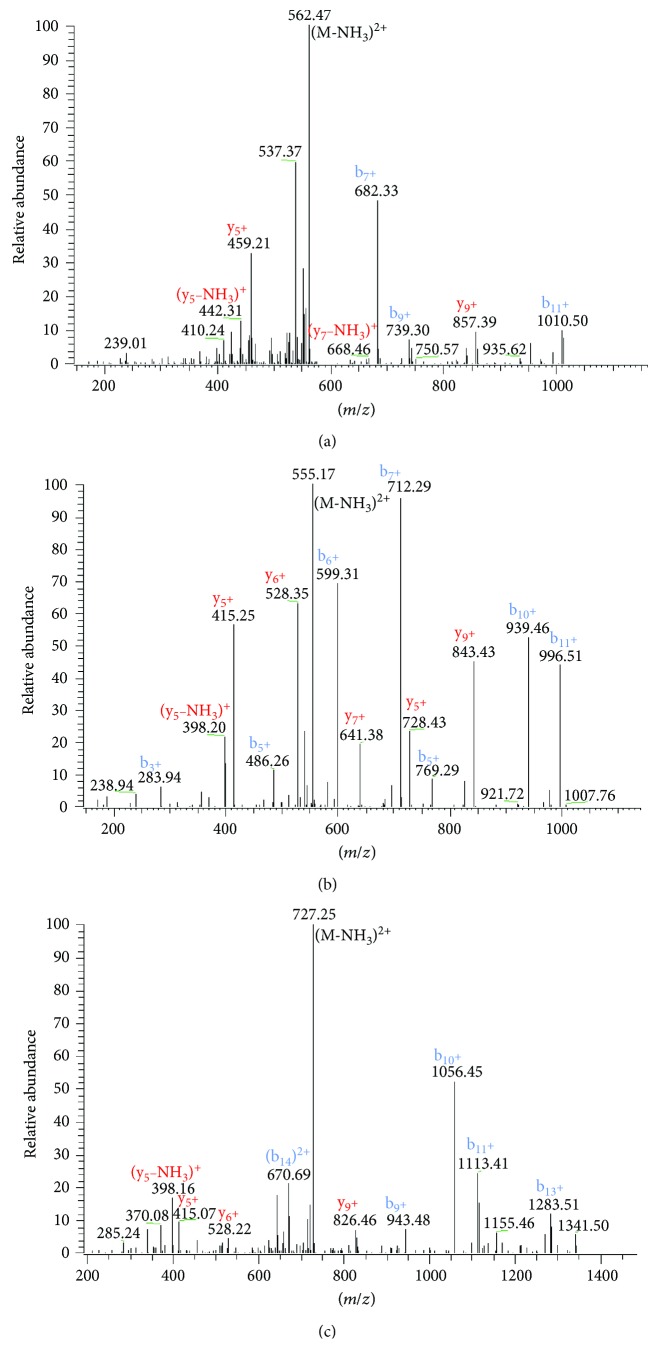
Annotated tandem mass (MS/MS) fragmentation spectrum of (a) Caeridin-1, (b) S5-Caeridin-1, and (c) Caeridin-a1. The observed *b* ions and *y* ions are labelled in blue and red, respectively.

The computed molecular masses of the deduced primary structures of Caeridin-1, S5-Caeridin-1, and Caeridin-a1 located within the open-reading frames of respective cloned cDNAs were used to detect each Caeridin within reversed-phase HPLC fractions as shown in Figure 4, which were subjected to mass spectrometric analysis. The primary structures of each Caeridin were confirmed by tandem mass spectrometric (MS/MS) fragmentation as shown in Figure 5 and Table 1. The existence of C-terminal amidation of each Caeridin was confirmed with the appropriated positioned G residue playing as the amide donor.

### 2.3. Secondary Structural Identification and Physicochemical Property Analysis of Caeridins

**Figure 6:**
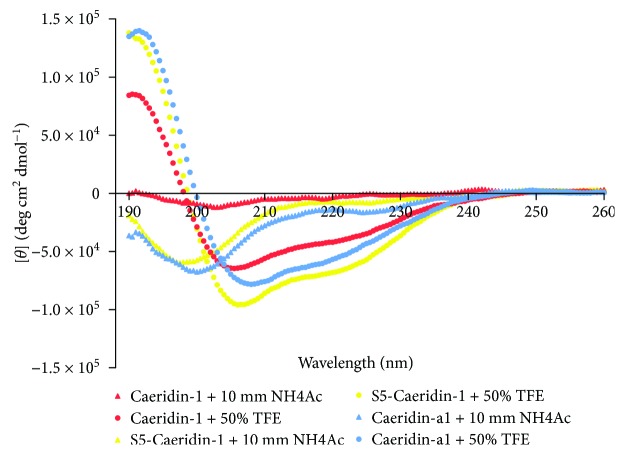
CD spectra under different conditions measured from 260 nm to 190 nm and plotted as mean residue ellipticity [θ] versus wavelength.

The purified synthetic replicates of Caeridins were subjected to secondary structural identification. From the CD spectra, these positive bands at 190 nm and double-negative bands at 208 nm and 222 nm in the 10 mM NH_4_Ac with 50% TFE solution indicated that Caeridin-1, S5-Caeridin-1, and Caeridin-a1 adopted a typical *α*-helical secondary structure in a membrane mimic environment (Figure 6). Multiple CD analysis methods were used and processed by the K2D3 online portal to measure the *α*-helicity of these Caeridins (Table 2) [13].

The newly discovered Caeridins together with the previously reported Caeridins were subjected to the analysis of physicochemical properties by the Heliquest Analysis online bioinformatic tool [14]. The hydrophobicity, hydrophobic moment (amphipathicity), net charge, and charged residues of these Caeridins were determined and are shown in Table 3.

### 2.4. Smooth Muscle Pharmacological Activities of Caeridins

**Figure 7:**
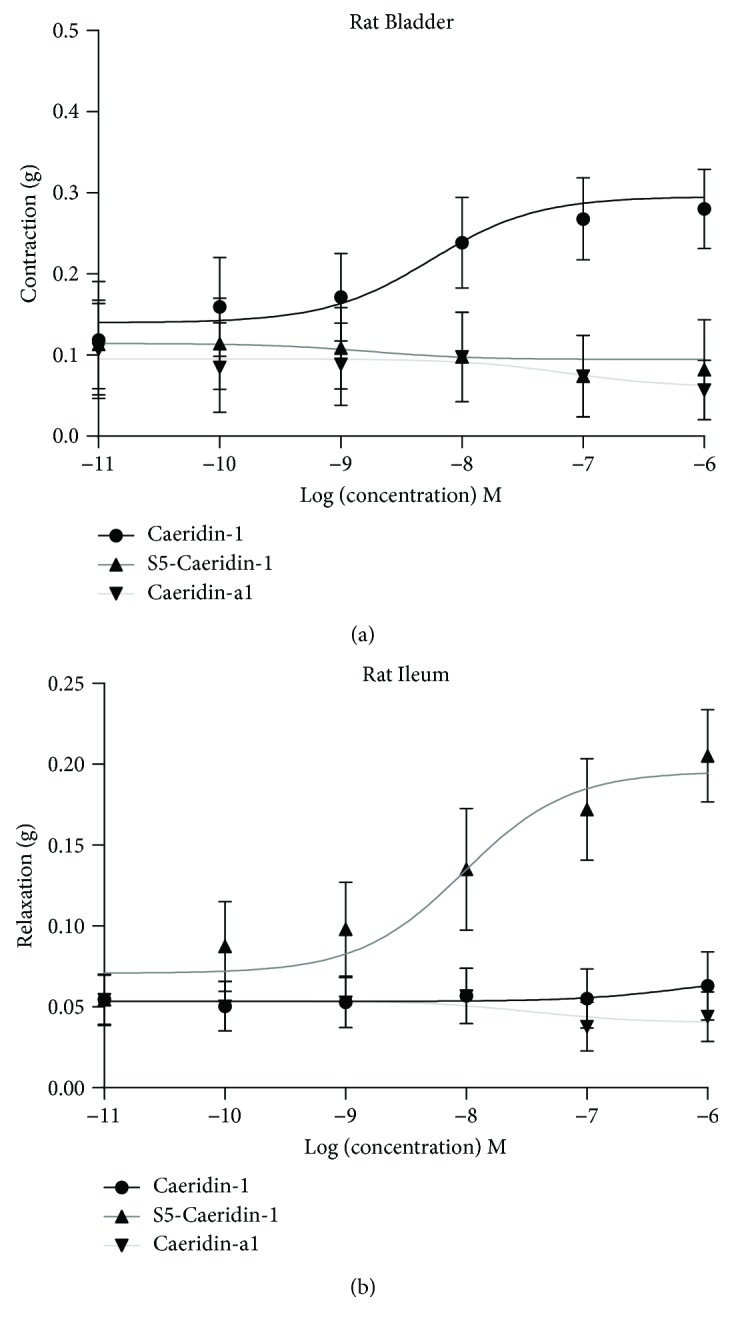
Dose-response curves of (a) synthetic Caeridins using rat urinary bladder smooth muscle preparations and (b) synthetic Caeridins on rat ileum smooth muscle preparations. Each point represents the mean and standard error of mean (SEM) of 5 determinations.

The purified synthetic replicates of Caeridins were subjected to smooth muscle pharmacological activity tests. Caeridin-1 was found to induce contractions on rat bladder smooth muscle, and S5-Caeridin-1 was found to have relaxation effects on rat ileum smooth muscle, at various concentrations, as shown in Figure 7. The half effective concentration (EC_50_) of Caeridin-1 inducing rat bladder smooth muscle contractions was 5.6 nM (5.6 × 10^−9^ M), and EC_50_ of S5-Caeridin-1 causing rat ileum smooth muscle relaxations was 9.5 nM (9.5 × 10^−9^ M). The maximum contraction effect of Caeridin-1 on bladder smooth muscle preparations was 0.3 g in tension change, and the maximum relaxant effect of S5-Caeridin-1 on ileum smooth muscle preparations was 0.2 g in tension change. Caeridin-a1 did not show any smooth muscle activities.

### 2.5. Antimicrobial Activities of Caeridins

The purified synthetic replicates of Caeridins were subjected to antimicrobial activity tests, with melittin and bradykinin as positive and negative controls, respectively. Melittin originally discovered from the venom of the honeybee (*Apis mellifera*) has potent antimicrobial activities. The inclusion of melittin as the positive peptide in the antimicrobial activity tests set a reference for the evaluation of the antimicrobial activity of the novel peptides [15–17]. Bradykinin derived from the frog skin extract of the European grass frog (*Rana temporaria*) is the counterpart to mammalian bradykinins with no obvious antimicrobial activities; thus, it is incorporated as the negative peptide in the antimicrobial activity tests [18–20]. Caeridin-a1 exhibited different levels of antimicrobial activities against the growth of the tested microorganisms (Table 4) and demonstrated the most potent antimicrobial activity against *Staphylococcus aureus* (*S. aureus*). It also inhibited the growth of antibiotic-resistant bacteria, methicillin-resistant *S. aureus* (MRSA), and *Enterococcus faecalis* (*E. faecalis*), but slightly less potent than that against *S. aureus*. It also had antimicrobial activities against the Gram-negative bacterium, *Escherichia coli* (*E. coli*) and the yeast, *Candida albicans* (*C. albicans*). Caeridin-1 and S5-Caeridin-1 did not demonstrate any antimicrobial activities on these tested microorganisms.

### 2.6. Membrane Permeability Studies

The novel antimicrobial peptide Caeridin-a1, melittin, and bradykinin were tested in the membrane permeability studies. Melittin is a canonical membrane interactive peptide, so it was included as the positive peptide [15–17]. Bradykinin was referred to as the negative peptide for it is known to be inactive on membrane interaction and permeabilisation [18–20].

#### 2.6.1. Membrane Permeability Studies on *S. aureus*

**Figure 8:**
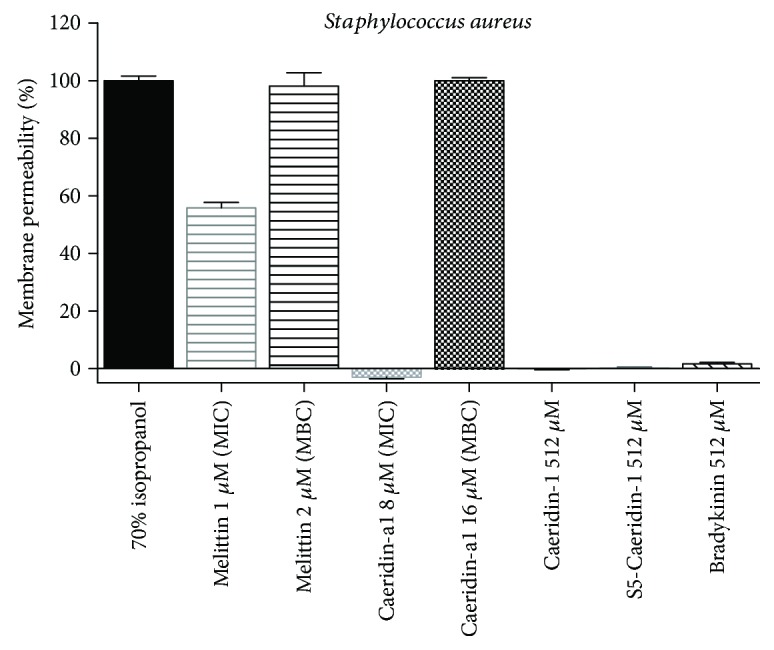
Membrane permeabilisation of *S. aureus* treated with melittin (MIC = 1 *μ*M and MBC = 2 *μ*M), Caeridin-a1 (MIC = 8 *μ*M and MBC = 16 *μ*M), Caeridin-1 (512 *μ*M), S5-Caeridin-1 (512 *μ*M), bradykinin (512 *μ*M), and 70% isopropanol for 2 h. The degree of membrane permeability was calculated in comparison with the maximum membrane permeabilisation control (70% isopropanol-treated). The error bars represent SEM of three independent experiments and each experiment with five replicates.

The peptide-induced permeabilisation of the cytoplasmic membranes of *S. aureus* was detected using SYTOX Green, a counterpart stain of nucleic acid and an indicator of dead cells. The cell membrane of *S. aureus* remained intact after being treated with the purified synthetic Caeridin-a1 (MIC) for 2 h, but became massively compromised after being treated with the purified synthetic Caeridin-a1 (MBC) for the same period of time, as shown in Figure 8. This phenomena suggested that Caeridin-a1 (MIC) failed to induce permeabilisation to the cytoplasmic membrane of *S. aureus* within the 2 h treatment. After the 2 h treatment, the positive peptide melittin produced approximately 50% membrane permeability at MIC (1 *μ*M) and 100% membrane permeability at MBC (2 *μ*M). The negative peptide bradykinin, Caeridin-1, and S5-Caeridin-1 barely showed membrane-permeabilising activity up to 512 *μ*M.

#### 2.6.2. Membrane Permeability Tests on *E. coli*

**Figure 9:**
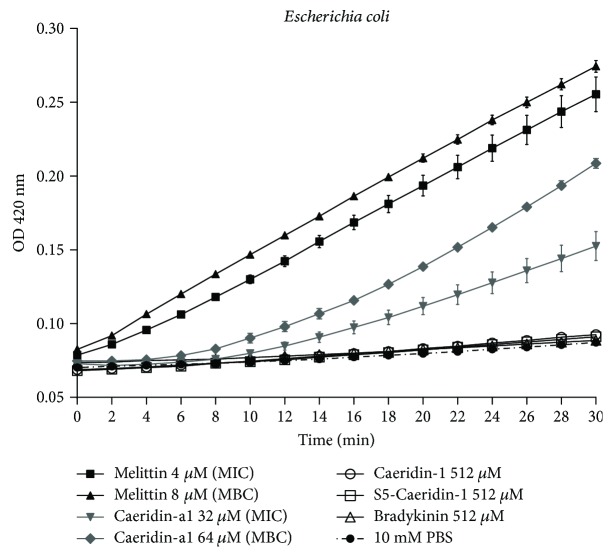
Membrane permeabilisation of *E. coli* treated with Caeridin-a1, Caeridin-1, S5-Caeridin-1, melittin, and bradykinin. The hydrolysis of ONPG generated by the release of cytoplasmic *β*-galactosidase of *E. coli* after treatment at MIC and MBC of Caeridin-a1, MIC and MBC of melittin, and maximum tested concentration of Caeridin-1, S5-Caeridin-1, and bradykinin was measured spectroscopically at 420 nm UV for 30 min. Each point represents mean absorbance and SEM of three independent experiments and each experiment with five replicates.

The peptide-induced membrane permeabilisation of *E. coli* was determined by the release of *β*-galactosidase activity into culture medium MHB (2% lactose) using ONPG as the substrate. As shown in Figure 9, the purified synthetic Caeridin-a1 (MIC and MBC) induced inner membrane permeabilisation within 30 min given that the ONPG entered the cytoplasm and was degraded by *β*-galactosidase, generating o-nitrophenol that produced an absorbance at 420 nm. The Caeridin-a1-induced membrane permeabilisation of *E. coli* was not as strong as the positive peptide melittin, evidenced by the higher absorbance at 420 nm, indicating an increased production of o-nitrophenol by ONPG hydrolysis. Caeridin-1, S5-Caeridin-1, and the negative peptide bradykinin failed to induce membrane permeabilisation of *E. coli*, as no absorbance increase was observed at 420 nm.

#### 2.6.3. Membrane Permeability Study on Human Microvascular Endothelial Cell Line, HMEC-1

**Figure 10:**
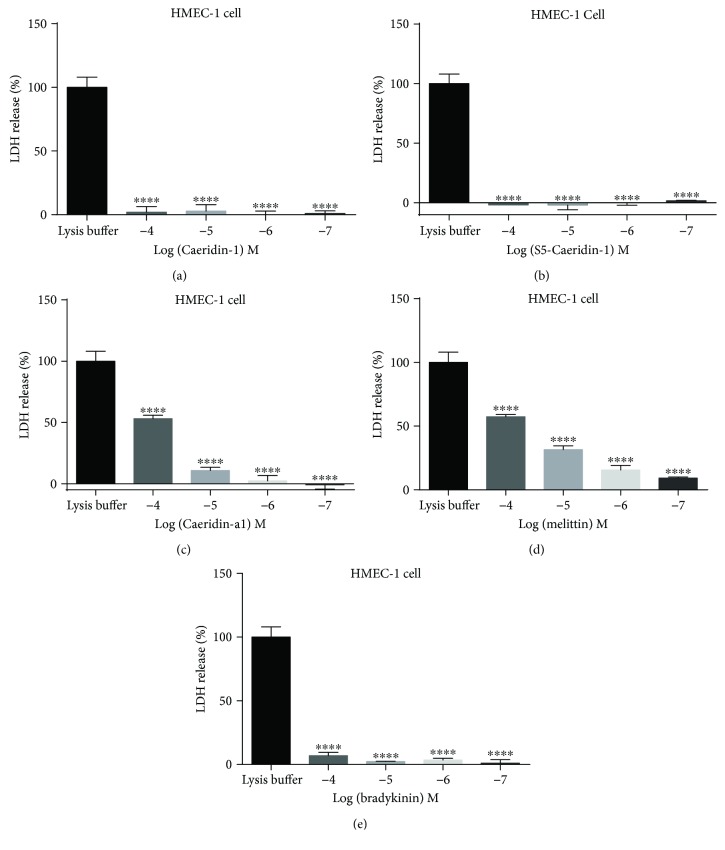
Membrane permeabilisation of human microvascular endothelial cell line, HMEC-1, reflected by the level of LDH released into the medium, after being treated by (a) Caeridin-1, (b) S5-Caeridin-1, (c) Caeridin-a1, (d) positive peptide melittin, and (e) negative peptide Bradykinin. The level of LDH release was calculated in comparison with the maximum LDH release control group (lysis buffer-treated)). ^∗∗∗∗^*p* < 0.0001 represents a significant change in the level of LDH release, compared with the maximum LDH release control group (lysis buffer-treated), using one-way analysis of variance followed by Dunnett's multiple comparisons tests. The error bar represents SEM of three independent experiment determinations and each experiment with triplicates.

The peptide-induced membrane permeabilisation of normal human cells was evaluated by the release of lactate dehydrogenase (LDH), a cytosolic enzyme, from the damaged membrane of the human microvascular endothelial cell line HMEC-1. After treatment with Caeridin-1, S5-Caeridin-1, and negative peptide bradykinin at concentrations of 10^−7^ M to 10^−4^ M, lower than 5% of membrane lysis was resulted (Figures 10(a), 10(b), and 10(e)). In comparision, Caeridin-a1-induced membrane permeabilisation of HMEC-1 cells was indicated by about 50% of LDH release at 100 *μ*M and approximately 10% LDH release at 10 *μ*M (Figure 10(c)). The positive peptide melittin showed membrane permeability in HMEC-1 cells at a concentration as low as 0.1 *μ*M, suggested by approximately 10% LDH release (Figure 10(d)). As the level of LDH released into the medium is indicative of cytotoxicity, Caeridin-1, S5-Caeridin-1, and negative peptide bradykinin showed weak cytotoxicity to HMEC-1 cells at 100 *μ*M, while Caeridin-a1 and positive peptide melittin showed significant cytotoxicity at 100 *μ*M. However, at the lower tested concentrations, no cytotoxicity was detected with Caeridins and negative peptide bradykinin.

### 2.7. Haemolysis Induced by Caeridins

**Figure 11:**
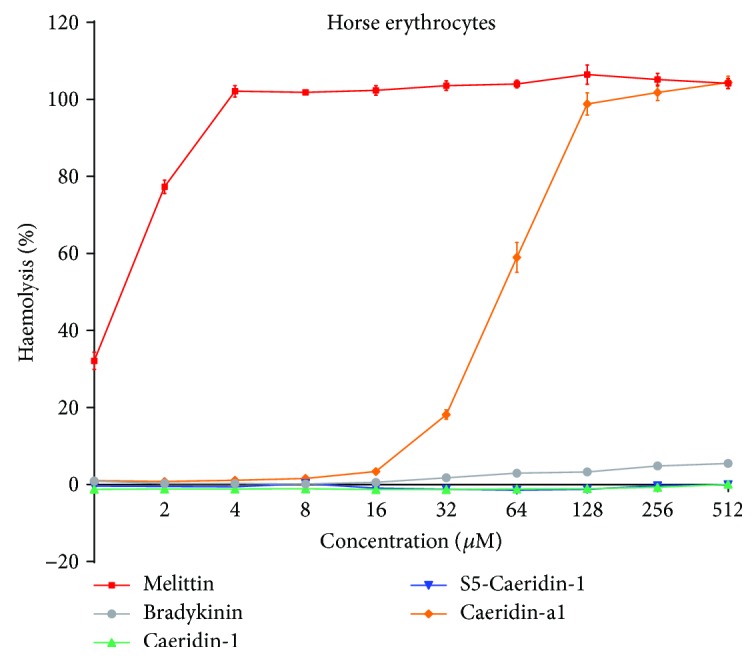
Haemolytic activities of synthetic Caeridin-1, S5-Caeridin-1, Caeridin-a1, melittin, and bradykinin. The level of haemolysis tested on horse red blood cells was calculated in comparison with the maximum haemolysis control (1% Triton X-100-treated). The error bar represents SEM of three independent experiment determinations and each experiment with five replicates.

Haemolytic activities of Caeridins, positive peptide melittin, and negative peptide bradykinin were studied using horse erythrocytes as shown in Figure 11. Melittin was incorporated as the positive peptide due to its known haemolytic activities. The amphibian bradykinin, the counterpart to mammalian Bradykinin, has so little haemolytic activities that it was included in the haemolysis test as the negative peptide [15–20]. The incorporation of melittin and bradykinin as positive and negative peptide provided evidence on the evaluation of the haemolytic activities of the novel peptides. Bradykinin, Caeridin-1, and S5-Caeridin-1 exhibited weak haemolytic activities at the tested concentrations, while Caeridin-a1 exhibited 18% haemolysis at 32 *μ*M, and positive peptide Melittin showed eminent haemolysis from 1 *μ*M.

## 3. Discussion

Peptides could offer multiple benefits over other drug treatments, such as higher potency, higher selectivity, a broader range of targets, potentially lower toxicity, lower accumulation in tissues, more abundance in chemical and biological diversity, discoverability at amino acid and nucleotide levels, and relatively low cost in production [2]. Thus, the future of many medicinal treatments could depend on the use of peptides and peptide-based drugs [21]. Frog skin secretions have been recognised as a valuable source of abundant bioactive peptides which are generally categorised and grouped into cytolytic peptides and pharmacologically active peptides [6]. Given their bioactive potential, there are now global efforts to minimise the observed global decline in frog populations through frog protection and environmental conservation strategies, along with the development of improved animal-friendly methods to conduct peptide discovery experiments from frog skin secretions [22–24].

Within this study, we have successfully constructed a cDNA library of the lyophilized skin secretion of the Australian White's tree frog, *L. caerulea*. *L. caerulea*, a member of the tree frog family Hylidae, was selected for analysis as it has been understudied to date and has multiple skin-defensive and regulatory compounds with significant pharmaceutical potentials that warrant further investigation [25, 26]. So far, three major peptide families have been identified from *L. caerulea*, namely, Caerins, Caeruleins, and Caeridins [9]. Caerins were found similar in primary structures with conserved -Gly-Leu- at the N-terminus, and they possess sufficient physiochemical characteristics to carry out antimicrobial activities [27]. Caeruleins, similar to the mammalian cholecystokinin-8 (CCK-8) and hexagastrin, were proven as powerful pharmacological peptides inducing gastrointestinal smooth muscle contractions and analgesic and hormonal activities [28]. Caeridins, unfortunately, were reported to have neither antimicrobial activities nor pharmacologically active activities [9]. From the constructed cDNA, we have consistently cloned three different preprocaeridin cDNAs by means of a 3′-RACE strategy, using a degenerate sense primer pool designed to a conserved nucleotide sequence in the 5′ untranslated region of homologous Caerin cDNA. These clones contained full-length open-reading frames of preprocaeridins. The signal peptide primary structures and nucleic acid sequences are highly conserved and some are identical, and the conserved trends extend to the acidic prosegments, but not very obviously conserved when it comes to mature peptide encoding regions, as shown in Figure 2. All these mature peptides deduced from cloned skin secretion cDNAs were found to have a C-terminal amidation in the corresponding reversed-phase HPLC fractions by the subsequent mass spectrometric analysis as shown in Figures 5 and 6. These mature peptides were subsequently compared with the protein database of the National Center of Biotechnology Information (NCBI), and it was found that one of these mature peptides was identical to Caeridin-1 with unknown transcriptomic information [9], here also called Caeridin-1. The peptide with two amino acids different from Caeridin-1 was called S5-Caeridin-1, and the final peptide with 80% identity to Caeridin-2 and Caeridin-3 was called Caeridin-a1.

The chemical synthetic replicates of Caeridin-1, S5-Caeridin-1, and Caeridin-a1 were tested through various bioassays. Antimicrobial activities of these Caeridins were screened on standard bacteria and drug-resistant bacteria given that peptides have previously shown potential to be developed as conventional antibiotic alternatives to unravel the global antimicrobial resistance. Smooth muscle is of great significance in physiological modulation; for example, the smooth muscle in the uterus helps in parturition, the smooth muscle in the bladder helps in uresis, the smooth muscle in arteries determines blood flow, and the smooth muscle in the digestive tract modulate nutrients and wastes mobility. Therefore, pharmacological activities of these Caeridins were tested on different rat smooth muscles.

Caeridin-1 and S5-Caeridin-1, deficient of physiochemical properties, were found to have no effects on membrane interaction and disruption and thus failed to show antimicrobial activities [29]. However, Caeridin-1 was found to mediate a contraction of rat urinary bladder smooth muscle with EC_50_ = 5.6 nM and a maximum contraction of 0.3 g in tension change with no haemolytic effects. S5-Caeridin-1 was found to induce a relaxation of rat ileum smooth muscle with EC_50_ = 9.5 nM and a maximum relaxation of 0.2 g in tension change with no haemolysis. The discovery of Caeridin-1 and S5-Caeridin-1 provided templates for developing urinary system and digestive system regulatory drugs, but the mechanisms of action need further investigations.

Caeridin-a1 was recognised to be neutral in charge, with a basic amino acid, -Lys-, and an acidic amino acid, -Asp-, and adopted *α*-helical conformation in the membrane-mimetic environment. Caeridin-a1 was found to be inhibitory against the growth of normal microorganisms, the Gram-positive bacterium, *S. aureus*, the Gram-negative bacterium, *E. coli*, and the yeast, *C. albicans*, as well as against MRSA and *β*-lactam-resistant bacterium *E*. *faecalis* which are hard to be controlled by conventional antibiotics and tend to cause more virulent infections. Antimicrobial susceptibility tests suggest that Caeridin-a1 showed better antimicrobial potency against the Gram-positive bacteria, *S. aureus*, MRSA, and *E. faecalis*, with MIC at 8 *μ*M, 16 *μ*M, and 32 *μ*M, respectively, but less potent against the Gram-negative bacterium, *E. coli*, with MIC at 32 *μ*M. As the membrane disruption is recognised as one of the mechanisms of antimicrobial actions of antimicrobial peptides, membrane permeability tests were performed on the standard Gram-positive bacterium (*S. aureus*) and Gram-negative bacterium (*E. coli*). The Caeridin-a1-induced membrane permeabilisation of *S. aureus* was monitored only at its MBC (16 *μ*M), indicating that Caeridin-a1 at its MIC = 8 *μ*M was not able to permeabilise the cytoplasmic membrane within 2 h. However, Caeridin-a1 induced membrane permeabilisation of *E. coli* within 30 min at both its MIC (32 *μ*M) and MBC (64 *μ*M) against *E. coli*. The significant membrane-disruptive effect of Caeridin-a1 was also observed in HMEC-1 cells at 100 *μ*M and was weakened with the decrease of the peptide concentration. These results suggest that the swift membrane permeability depends on the administrated concentration of Caeridin-a1. Caeridin-a1 at its higher concentrations could rapidly and unspecifically interact and disrupt the cytoplasmic membrane leading to cell lysis, but Caeridin-a1 at its lower concentrations may perform antimicrobial activities by other mechanisms or may need longer time to induce membrane permeabilisation. Caeridin-a1 was also found to induce haemolysis at higher concentrations, whereby 18% haemolysis was detected at 32 *μ*M, which consequently decreases its potential therapeutic value. However, the size of Caeridin-a1 is relatively small with only 15 amino acid residues, and therefore, Caeridin-a1 could be used as a template for developing smaller antimicrobial peptides after rational structural modification, which may be of interest to the pharmaceutical industry due to reduced cost of development and easier manufacturing processes.

Although the Caeridins discovered in this study share a similar amino acid composition and structural elements with each other and with the Caeridins reported previously, the bioactivities they exhibited are different. Only Caeridin-a1 was found to demonstrate potent antimicrobial and haemolytic effects, but Caeridin-1, S5-Caeridin-1, and other reported Caeridins were not. This could be explained by the variation in the membrane-disruptive action of peptides. The Gram-positive bacterial cell walls contain anionic teichoic acids and lipoteichoic acids. The anionic lipopolysaccharides are widely distributed in the outer leaflet of the outer membrane of Gram-negative bacteria. The negatively charged glycosaminoglycans and gangliosides are composed in mammalian cells exterior to the cytoplasmic membrane. Firstly, amphipathic peptides with positively charged residues are attracted to the vicinity of the cell surface by electrostatic actions. Secondly, peptides attach to the cell membrane via electrostatic and hydrophobic interactions. Then they undergo conformational transition to bind with the membrane and form transmembrane pores/channels or translocate phospholipid bilayers, thus destroying the membrane integrity. Because of the abundance of the acidic phospholipids, such as cardiolipin and phosphatidylglycerol, and the absence of sterol, in the cytoplasmic membrane of bacteria, amphipathic peptides with cationic residues are able to attach to the bacterial membrane by both electrostatic interaction and hydrophobic interaction. However, the mammalian cell membranes only have acidic phospholipids at the inner facet of the phospholipid bilayers, suggesting that peptides are more able to interact with the mammalian cell membrane by hydrophobic interaction. In addition to phospholipids, the numerous sterols embedded in the bilayers impede the peptide from better binding with the membrane and triggering further membrane disruption, which is the same on red blood cells. The cationic residue, -Lys-, in the amino acid composition of Caeridin-a1 may facilitate the peptide being better attracted to the cell surface and interacting with the cell membrane by electrostatic effects, though the net charge of Caeridin-a1 was 0 due to the presence of the anionic residue, -Asp-. In addition to electrostatic interaction, the high amphipathicity and hydrophobicity of Caeridin-a1 may also promote the peptide to bind to the cell membrane by hydrophobic interaction. Then, Caeridin-a1 may conform to an *α*-helical structure to bind with the membrane and form transmembrane pores, thus destroying the membrane. Therefore, Caeridin-a1 was able to disrupt the non-sterol-protected bacterial cell membrane at lower concentrations, and sterol/cholesterol-protected mammalian cell membrane and red blood cell membrane at higher concentrations, showing antimicrobial effects on the tested microorganisms, cytotoxic effects on HMEC-1 cells, and haemolytic effects on the horse erythrocytes. Regarding the other Caeridins, as shown in Table 3, the net charge of Caeridin-1, S5-Caeridin-1, Caeridin-2-4, and Caeridin-7.1 is −1 due to the presence of an acidic residue, -Asp-, and the net charge of Caeridin-5 and Caeridin-6 is zero because of the lack of charged residues. Therefore, these Caeridins are expected to fail to undergo electrostatic attraction and interaction with the membrane. They showed no membrane disruption in the tested microorganisms, mammalian cells, and horse red blood cells. Although no antimicrobial effects were detected, Caeridin-1 and S5-Caeridin-1 were presumed to be mediated by the G protein-coupled receptors to induce contraction and relaxation of smooth muscle preparations with no haemolysis and cytotoxicity. However, Caeridin-a1 with strong membrane disruptive activity would also cause damage to smooth muscle cell membrane; thus, no smooth muscle activities were found with Caeridin-a1.

The discovery of Caeridin-1, S5-Caeridin-1, and Caeridin-a1 reinforced the multiple bioactivities of the Caeridin peptide family. Although they are very similar with other reported Caeridins, certain amino acid mutations were identified. It is these small mutations in amino acid sequences and compositions that affect physicochemical characteristics and functioning structures and lead to big differences in bioactivities. Therefore, optimal amino acid sequence selections from frog skin secretions lay a foundation for peptide discovery and development.

## 4. Materials and Methods

### 4.1. Collection of Frog Skin Secretions

The adult Australian frogs (*n* = 4), *L. caerulea*, were captive bred and kept for three months before the collection of their skin secretions. Their defensive skin secretions were collected from their dorsal skin by mild transdermal electrical stimulation according to the technique reported by Tyler et al. [30], then lyophilised and stored at −20°C prior to analysis. Sampling of skin secretion was performed by Mei Zhou under UK Animal (Scientific Procedures) Act 1986, project license PPL 2800, issued by the Department of Health, Social Services and Public Safety, Northern Ireland. Procedures had been vetted by the IACUC of Queen's University Belfast and approved on 19 February 2016.

### 4.2. “Shotgun” Cloning of Novel Caeridin Precursor-Encoding cDNAs from the Skin Secretion-Derived cDNA Library of *L. caerulea*

The cDNA library of *L. caerulea* was established through the reverse transcription of the mRNA. The mRNA was extracted from the lyophilised skin secretion by using Dynabeads® mRNA DIRECT™ Kit (Life Technologies, Oslo, Norway). The extracted mRNA was reverse transcribed to synthesise the first-strand cDNA by following the instructions described by the BD SMART™ RACE cDNA Amplification Kit (Clontech, Palo Alto, CA, USA) to establish the cDNA library. The cDNA library was subjected to 3′-RACE (rapid amplification of cDNA ends) procedures to obtain full-length preprocaeridin nucleic acid sequence data with a SMART-RACE Kit (Clontech, Palo Alto, CA, USA) according to the manufacturer, which involved a NUP primer supplied with the kit and a degenerate sense primer pool (S1: 5′-GVCCTTGTAAAGACCAAVCATGGCTT-3′) designed to a segment of the 5′-untranslated region of Caerin cDNAs cloned from the skin secretion of *L. caerulea* (EMBL accession Number AY218778-82). This approach was applied as the segment of the 5′-untranslated region employed was found to be highly conserved among the homologous peptide cDNAs [31]. An additional advantage of this strategy was that the entire open-reading frame of these transcripts could be deduced through a single 3′-RACE procedure. This PCR cycling procedure was carried out as follows: initial denaturation step: 60 s at 94°C; 40 cycles of amplification: denaturing for 30 s at 94°C; primer annealing for 30 s at 61°C; and strand extension for 180 s at 72°C. RACE-PCR products were analysed by DNA agarose gel electrophoresis, purified, and cloned in a pGEM®-T Easy vector system (Promega Corporation, Southampton, UK), and the selected samples were sequenced by an ABI 3730 DNA automated analyser (Applied Biosystems, Foster City, CA, USA). The nucleic acid sequences were translated into amino acid sequences by the ExPASy Translate Tool online portal. The deduced preprocaeridins were analysed using the Blast Alignment Search Tool (BLAST) of the National Centre for Biotechnology Information (NCBI) by comparing to peptides with known amino acid sequences in the protein database. Alignments of similar regions were established by MEGA 6. The nucleotide sequences of the cDNA encoding the novel Caeridins were registered with the European Nucleotide Archive (ENA) browser at http://www.ebi.ac.uk/embl/genomes/submission/.

### 4.3. Chromatographic Isolation and Primary Structural Characterisation of Novel cDNA-Deduced Caeridins from the Skin Secretion of *L. caerulea*

The lyophilised skin secretion of *L. caerulea* dissolved in solution A (99.95% H_2_O, 0.05% trifluoroacetic acid (TFA) was fractionated by reversed-phase HPLC using a Waters Binary pump HPLC system (Waters, Milford, MA, USA) fitted with an analytical column (Jupiter, C5, 300 Å, 5 *μ*m, 250 mm × 4.6 mm, Phenomenex, Macclesfield, Cheshire, UK) and eluted with a linear gradient formed from 100% solution A (99.95% H_2_O, 0.05% TFA) to 100% solution B (80.00% acetonitrile, 19.95% H_2_O, 0.05% TFA) in 240 min at a flow rate of 1000 *μ*l/min. Fractions were collected at each minute, and the effluent was continuously detected at *λ* = 214 nm. The dead volume between column and fraction collector was minimal (20 *μ*l).

The molecular masses of peptides in each fraction were analysed by matrix-assisted laser desorption/ionisation time-of-flight mass spectrometry (MALDI-TOF MS) on a linear time-of-flight Voyager DE mass spectrometer (Voyager DE, Perspective Biosystems, Foster City, CA, USA) in a positive detection mode using *α*-cyano-4-hydroxycinnamic acid matrix to construct a mass spectral library of the *L. caerulea* skin secretion peptide. The instrument was calibrated in the range of 1–4 kDa, and the accuracy of mass determinations was ±0.1%. The computed molecular masses of predicted mature Caeridins deduced from encoded cDNA were used to interrogate the mass spectral library to identify the putative Caeridins. The skin secretion fractions containing the putative Caeridins diluted in solution A (99.90% H_2_O, 0.10% formic acid) were pumped directly onto an analytical HPLC column (Jupiter, C18, 300 Å, 5 *μ*m, 150 mm × 4.6 mm, Phenomenex, Macclesfield, Cheshire, UK) connected to an LCQ Fleet ESI ion-trap mass spectrometer (Thermo Fisher, San Jose, CA, USA) and eluted from 100% solution A (99.90% H_2_O, 0.10% formic acid) to 100% solution B (19.90% H_2_O, 80.00% acetonitrile, and 0.10% formic acid) in 135 min at a flow rate of 20 *μ*l/min. Mass analysis was performed in a positive ion mode with acquired spectra in the range of 500–2000 *m*/*z* with >50% relative intensity during HPLC-MS. Parameters for electrospray ionisation ion-trap mass spectrometry (ESI/MS) were spray voltage +4.5 kV, drying gas temperature 320°C, drying gas flow 200 *μ*l/min, and maximum accumulation time (ion trap) 350 ms. The first mass analysis was performed in full-scan mode, then peptide ions with >50% relative intensity were selected for fragmentation by collision-induced dissociation (CID), to generate *b* and *y* ions detected in a second mass analysis. The instrument was controlled by Xcalibur software (Thermo Fisher, San Jose, CA, USA), and data analysis was performed using Proteome Discoverer 1.0 (Thermo Fisher, San Jose, CA, USA). Sequest™ algorithm was employed to compare the acquired fragment ion profiles with the theoretical fragment ions generated from a FASTA database specific for this species built by “shotgun” cloning (as described above) to confirm the amino acid sequences of individual Caeridins.

### 4.4. Solid-Phase Peptide Synthesis

The novel Caeridins, positive peptide Melittin, and negative peptide Bradykinin were synthesised by solid-phase Fmoc chemistry using a Tribute automated solid phase peptide synthesiser 4 (Protein Technologies, Tucson, AZ, USA). After cleavage from resin and deprotection, each crude peptide was obtained. The amino acid sequences of these peptides are summarised in Table 5. The authenticity of the synthetic peptides was established by HPLC purification and confirmed by MALDI-TOF (Voyager DE, Perspective Biosystems, Foster City, CA, USA) and an LCQ Fleet electrospray ion-trap mass spectrometer (Thermo Fisher Scientific, San Francisco, CA, USA).

### 4.5. Secondary Structural Determination by Circular Dichroism (CD)

CD spectra were obtained at 20°C using a 1 mm high-precision quartz cell (Hellma Analytics, Essex, UK) with a JASCO J-815 CD spectrometer (Jasco, Essex, UK). The measurement range was from 260 nm to 190 nm at a scanning speed of 200 nm/min. The bandwidth was 1 nm, and the data pitch was 0.5 nm. Caeridins were, respectively, dissolved in 10 mM NH_4_Ac or 10 mM NH_4_Ac with 50% TFE to reach a final concentration of 100 *μ*M. The CD spectra were analysed by a K2D3 online portal programme [13].

### 4.6. Rat Smooth Muscle Tests

Female Wistar rats (250–300 g) were euthanized by CO_2_ asphyxiation followed by cervical dislocation under appropriate Home Office (UK) animal licenses. Full urinary bladder and ileum were carefully removed from the abdomen, then placed in cold Kreb's solution (118 mM NaCl, 4.7 mM KCl, 25 mM NaHCO_3_, 1.15 mM NaH_2_PO_4_, 2.5 mM CaCl_2_, 1.1 mM MgCl_2_, and 5.6 mM glucose), equilibrated with a carbogen mixture (95% O_2_, 5% CO_2_) [32]. The prepared smooth muscles linked with transducers were placed in 2 ml capacity organ baths with Kreb's solution flowing through at 2 ml/min and maintained at 37°C with constant bubbling of carbogen gas mixture (95% O_2_, 5% O_2_) and equilibrated for 1 h prior to analysis. Then the viability of these smooth muscle preparations was tested by 60 mM KCl. Caeridins were tested at sequential molar concentrations in the range from 10^−6^ M to 10^−11^ M. Dose-response curves were depicted for each Caeridin on individual smooth muscle preparations (*n* = 5). Changes in tension of the smooth muscle preparations were detected by pressure transducers connected to a PowerLab System (ADInstruments Pty Ltd.). Data were recorded and calculated to obtain the means of responses and standard error of means (SEM). The dose-response curve of each Caeridin was established using best-fit algorithms through the data analysis package provided by GraphPad Prism (Version 6.0; GraphPad Software Inc., San Diego, CA, USA), and the comparative EC_50_ value for each peptide was depicted.

### 4.7. Antimicrobial Tests

The antimicrobial activities were tested by the broth microdilution method and evaluated by the determination of MIC values against different tested microorganisms, the Gram-positive bacteria, *S. aureus* (NCTC 10788), *E. faecalis* (NCTC12697), and MRSA (NCTC 12493); the Gram-negative bacterium, *E. coli* (NCTC 10418); and the yeast, *C. albicans* (NCYC 1467). The microorganisms were cultured to their log phase in MHB and diluted to 5 × 10^5^ CFU/ml then subsequently treated with peptide solutions of gradient concentrations. After incubation at 37°C overnight, the growth of microorganisms was detected at a wavelength of 550 nm using a Synergy HT plate reader (Biolise BioTek ELx808, Winooski, VT, USA). The MICs were determined as the lowest concentration of peptide where growth was detectable.

### 4.8. Cell Membrane-Permeable Tests

The cell membrane permeability of *S. aureus* was tested with SYTOX Green Nucleic Acid Stain (Life Technologies, Carlsbad, CA, USA). The log-phase *S. aureus* was pelleted and suspended in 5% TSB in 0.85% NaCl solution to achieve 1 × 10^8^ CFU/ml cells which was then treated with peptide solutions at 37°C for 2 h. The maximum membrane-compromised *S. aureus* was produced by 70% isopropanol treatment. Subsequently, cells were mixed with 5 *μ*M SYTOX Green stain and incubated in the dark for 10 min. The fluorescence was measured in a 37°C Synergy HT plate reader (Biolise BioTek ELx808, Winooski, VT, USA) at excitation and emission wavelengths of 480 nm and 528 nm, respectively. The membrane permeability was calculated as

(1) $$\begin{array}{c} Membrane\;permeability\left(\%\right)=\frac{\left(F-F_{0}\right)}{\left(F_{1}-F_{0}\right)}\times 100\%, \end{array}$$

where *F* represents the processed fluorescence intensity with peptide treatments, *F*_0_ represents the processed fluorescence intensity of untreated *S. aureus*, and *F*_1_ represents the processed fluorescence intensity in the maximum membrane-compromised *S. aureus* (70% isopropanol-treated). The fluorescence intensities of background were subtracted prior to calculation.

The membrane permeabilsation of *E.coli* was determined by measuring the release of *β*-galactosidase activity from *E. coli* into the culture medium with ONPG as the substrate. The log-phase *E. coli* was grown in MHB medium containing 2% lactose at 37°C, and the cells were pelleted. The cell pellets were suspended in 10 mM PBS (pH 7.4) containing 1.5 mM ONPG and diluted to 1 × 10^8^ CFU/ml. Aliquots of cells were incubated with different concentrations of peptides at 37°C. Permeabilisation was measured at 420 nm every 2 min from 0 to 30 min, which reflects ONPG influx into the cells working as an indicator of the permeability of the tested peptides.

The membrane permeabilisation of the human microvascular endothelial cell line, HMEC-1, was evaluated using the Pierce LDH Cytotoxicity Assay Kit (Thermo Fisher Scientific, San Francisco, CA, USA). HMEC-1 cells were cultured in MCDB-131 medium (Life Technology, Paisley, UK) with 10% FBS, 10 ng/ml epidermal growth factor (EGF) (Life Technology, Paisley, UK), 10 mM L-glutamine (Life Technology, Paisley, UK), and 1% penicillin-streptomycin (Sigma-Aldrich, St. Louise, MO, USA), at 37°C under 5% CO_2_. Cells were seeded in a 96-well plate at 5000 cells per well and cultured overnight to 80% confluency. Then, the cells were treated with different peptide solutions of gradient concentrations from 10^−7^ M to 10^−4^ M for 24 h at 37°C, 5% CO_2_, and H_2_O-treated cells were made as the spontaneous LDH release control. Lysis buffer was added to cells and incubated for 45 min at 37°C, 5% CO_2_, to produce the lysis buffer-treated cells which were referred to as maximum LDH release control. Then 50 *μ*l of each sample supernatant was transferred to a 96-well flat-bottom plate in triplicate wells, to which 50 *μ*l of reaction mixture was added, incubated, and protected from light at room temperature for 30 min. After addition of 50 *μ*l Stop Solution to each sample well and removal of bubbles present in sample wells, the absorbance at 490 nm and 680 nm was measured. The absorbance values at 680 nm were subtracted from the absorbance values at 490 nm before calculation of the level of LDH release (cytotoxicity). The level of LDH release was calculated as

(2) $$\begin{array}{c} LDH\;release\;\left(\%\right)=\frac{\left(L-L_{0}\right)}{\left(L_{1}-L_{0}\right)}\times 100\%, \end{array}$$

where *L* refers to sample-treated LDH activity, *L*_0_ to spontaneous (H_2_O-treated) LDH activity, and *L*_1_ to maximum (lysis buffer-treated) LDH activity.

Data analysis was performed using one-way analysis of variance followed by Dunnett's multiple comparison tests with maximum LDH release control in GraphPad Prism (Version 6.0; GraphPad Software Inc., San Diego, CA, USA).

### 4.9. Haemolytic Tests

The haemolysis of Caeridins was tested on prepared defrinated horse red blood cells (TCS Biosciences Ltd., Buckingham, UK) by incubating equal volumes of peptide solutions with erythrocyte suspensions at 37°C for 2 H. Maximum haemolysis control was included by incubating equal volumes of 1% Triton X-100 (Sigma-Aldrich, St. Louis, MO, USA) with erythrocyte suspensions. Lysis of erythrocytes was detected by the measurement of supernatant absorbance at a wavelength of 550 nm using a Synergy HT plate reader (Biolise BioTek ELx808, Winooski, VT, USA). The level of haemolysis was as calculated as

(3) $$\begin{array}{c} Haemolysis\;\left(\%\right)=\frac{\left(A-A_{0}\right)}{\left(A_{1}-A_{0}\right)}\times 100\%, \end{array}$$

where *A* represents the absorbance with peptide treatments, *A*_0_ represents the average absorbance in untreated erythrocytes, and *A*_1_ represents the average absorbance in the maximum haemolysis control (1% Triton X-100-treated).

## 5. Conclusions

The discovery of Caeridin-1, S5-Caeridin-1, and Caeridin-a1 not only contributed templates for drug design but also provided evidence that peptides from frog skin secretions possess bioactive properties and uphold expectations that peptides derived from natural resources may contribute to the clinical development of novel therapeutics.

## Figures and Tables

**Table 1:** Predicted singly and doubly charged *b* ions and *y* ions arising from MS/MS fragmentation of (a) Caeridin-1, (b) S5-Caeridin-1, and (c) Caeridin-a1. Actual fragment ions observed following MS/MS fragmentation are indicated in bold and italic.

Number 1	b(1+)	b(2+)	Seq.	y(1+)	y(2+)	Number 2
			(a)			
1	58.03	29.52	G			12
2	**171.11**	86.06	L	1083.68	*542.34*	11
3	**284.20**	142.60	L	*970.59*	485.80	10
4	**399.27**	**200.12**	D	*857.39*	*429.26*	9
5	**456.25**	**228.63**	G	*742.48*	*371.74*	8
6	**569.33**	**285.17**	L	*685.46*	*343.23*	7
7	**682.33**	**341.71**	L	*572.38*	*286.69*	6
8	**739.30**	**370.22**	G	*459.21*	230.15	5
9	**840.58**	420.74	T	*402.27*	201.64	4
10	**953.41**	**477.29**	L	*301.22*	151.12	3
11	**1010.50**	**505.80**	G	*188.05*	94.57	2
12			L-Amidated	131.12	66.06	1

			(b)			
1	58.03	29.52	G			12
2	**171.11**	86.06	L	1069.66	*535.33*	11
3	**283.94**	142.60	L	*956.58*	478.79	10
4	**399.22**	200.12	D	*843.43*	*422.25*	9
5	**486.26**	243.63	S	*728.43*	364.74	8
6	**599.31**	300.17	L	*641.38*	321.22	7
7	**712.42**	356.72	L	*528.35*	264.68	6
8	**769.29**	385.23	G	*415.25*	208.14	5
9	**826.47**	413.74	G	*358.25*	179.63	4
10	**939.46**	**470.28**	L	*301.22*	151.12	3
11	**996.51**	**498.79**	G	*188.14*	94.57	2
12			L-Amidated	131.12	66.06	1

			(c)			
1	58.03	29.52	G			15
2	**171.11**	86.06	L	1413.88	*707.44*	14
3	**318.18**	160.09	F	*1300.79*	*650.90*	13
4	**433.21**	217.61	D	*1153.73*	577.67	12
5	**546.29**	274.15	I	*1038.70*	519.86	11
6	**645.36**	323.68	V	*925.62*	463.31	10
7	**702.38**	352.19	G	*826.46*	413.78	9
8	**830.48**	415.7	K	*769.53*	385.27	8
9	**943.48**	472.3	L	*641.38*	321.22	7
10	**1056.45**	528.8	L	*528.22*	264.68	6
11	**1113.41**	557.3	G	*415.07*	208.14	5
12	**1169.48**	585.9	G	*358.25*	179.63	4
13	**1283.51**	**642.2**	L	*301.22*	151.12	3
14	**1340.79**	**670.7**	G	*188.05*	94.57	2
15			L-Amidated	131.12	66.06	1

**Table 2:** Secondary structures of Caeridin-1, S5-Caeridin-1, and Caeridin-a1 deduced from CD spectra by K2D3 programme.

Peptide	Method	*α*	*β*
Caeridin-1	K2D3	86.68%	0.02%
S5-Caeridin-1	K2D3	94.98%	0.01%
Caeridin-a1	K2D3	95.13%	0.02%

*α* = helices; *β* = strands.

**Table 3:** Physicochemical properties of Caeridins determined by Heliquest.

Peptides	Hydrophobicity	Hydrophobic moment	Net charge	Charged residues
Caeridin-1	0.807	0.417	−1	ASP 1
S5-Caeridin-1	0.783	0.413	−1	ASP 1
Caeridin-a1	0.77	0.477	0	ASP 1, LYS 1
Caeridin-2	0.751	0.45	−1	ASP 1
Caeridin-3	0.735	0.489	−1	ASP 1
Caeridin-4	0.725	0.421	−1	ASP 1
Caeridin-5	0.841	0.369	0	none
Caeridin-6	0.878	0.377	0	none
Caeridin-7.1	0.757	0.615	−1	ASP 1

**Table 4:** Minimum inhibitory concentration (MIC) and minimum bactericidal concentration (MBC) of Caeridins, melittin, and bradykinin against different tested microorganisms.

Peptides	MIC/MBC (*μ*M)				MIC/MFC (*μ*M)
	*S. aureus* NCTC10788	MRSA NCTC12493	*E. faecalis* NCTC12697	*E. coli* NCTC10418	*C. albicans* NCYC1467
Caeridin-a1	8/16	16/32	32/64	32/64	32/64
S5-Caeridin-1	>512/>512	>512/>512	>512/>512	>512/>512	>512/>512
Caeridin-1	>512/>512	>512/>512	>512/>512	>512/>512	>512/>512
Melittin	1/2	2/4	2/4	4/8	1/8
Bradykinin	>512/>512	>512/>512	>512/>512	>512/>512	>512/>512

**Table 5:** The amino acid compositions of novel Caeridins, positive peptide melittin, and negative peptide bradykinin.

Peptide	Amino acid sequence
Caeridin-1	H_2_N-GLLDGLLGTLGL-CONH_2_
S5-Caeridin-1	N_2_H-GLLDSLLGGLGL-CONH_2_
Caeridin-a1	H_2_N-GLFDIVGKLLGGLGL-CONH_2_
Melittin	H_2_N-GIGAVLKVLTTGLPALISWIKRKRQQ- CONH_2_ [15–17]
Bradykinin	H_2_N-RPPGFSPFR-COOH [18–20]

## Data Availability

The data used to support the findings of this study are available from the corresponding author upon request.

## Abbreviations

HPLC: High-performance liquid chromatography
MIC: Minimum inhibitory concentration
MBC: Minimum bactericidal concentration
CID: Collision-induced dissociation
MALDI-TOF MS: Matrix-assisted laser desorption/ionisation time-of-flight mass spectrometry
ESI/MS: Electrospray ionisation ion-trap mass spectrometry.
